# Use of personalised risk-based screening schedules to optimise workload and sojourn time in screening programmes for diabetic retinopathy: A retrospective cohort study

**DOI:** 10.1371/journal.pmed.1002945

**Published:** 2019-10-17

**Authors:** Andreas Ochs, Stuart McGurnaghan, Mike W. Black, Graham P. Leese, Sam Philip, Naveed Sattar, Caroline Styles, Sarah H. Wild, Paul M. McKeigue, Helen M. Colhoun, on behalf of the Scottish Diabetes Research Network Epidemiology Group and the Diabetic Retinopathy Screening Collaborative

**Affiliations:** 1 Institute of Genetics and Molecular Medicine, University of Edinburgh, Edinburgh, United Kingdom; 2 Diabetic Retinopathy Screening Collaborative, NHS Highland, Inverness, United Kingdom; 3 Ninewells Hospital, Dundee, United Kingdom; 4 Grampian Diabetes Research Unit, Diabetes Centre, Aberdeen Royal Infirmary, Aberdeen, United Kingdom; 5 British Heart Foundation Glasgow Cardiovascular Research Centre, University of Glasgow, Glasgow, United Kingdom; 6 Queen Margaret Hospital, Dunfermline, United Kingdom; 7 Usher Institute of Population Health Sciences and Informatics, University of Edinburgh, Edinburgh, United Kingdom; Chinese University of Hong Kong, CHINA

## Abstract

**Background:**

National guidelines in most countries set screening intervals for diabetic retinopathy (DR) that are insufficiently informed by contemporary incidence rates. This has unspecified implications for interval disease risks (IDs) of referable DR, disparities in ID between groups or individuals, time spent in referable state before screening (sojourn time), and workload. We explored the effect of various screening schedules on these outcomes and developed an open-access interactive policy tool informed by contemporary DR incidence rates.

**Methods and findings:**

Scottish Diabetic Retinopathy Screening Programme data from 1 January 2007 to 31 December 2016 were linked to diabetes registry data. This yielded 128,606 screening examinations in people with type 1 diabetes (T1D) and 1,384,360 examinations in people with type 2 diabetes (T2D). Among those with T1D, 47% of those without and 44% of those with referable DR were female, mean diabetes duration was 21 and 23 years, respectively, and mean age was 26 and 24 years, respectively. Among those with T2D, 44% of those without and 42% of those with referable DR were female, mean diabetes duration was 9 and 14 years, respectively, and mean age was 58 and 52 years, respectively. Individual probability of developing referable DR was estimated using a generalised linear model and was used to calculate the intervals needed to achieve various IDs across prior grade strata, or at the individual level, and the resultant workload and sojourn time. The current policy in Scotland—screening people with no or mild disease annually and moderate disease every 6 months—yielded large differences in ID by prior grade (13.2%, 3.6%, and 0.6% annually for moderate, mild, and no prior DR strata, respectively, in T1D) and diabetes type (2.4% in T1D and 0.6% in T2D overall). Maintaining these overall risks but equalising risk across prior grade strata would require extremely short intervals in those with moderate DR (1–2 months) and very long intervals in those with no prior DR (35–47 months), with little change in workload or average sojourn time. Changing to intervals of 12, 9, and 3 months in T1D and to 24, 9, and 3 months in T2D for no, mild, and moderate DR strata, respectively, would substantially reduce disparity in ID across strata and between diabetes types whilst reducing workload by 26% and increasing sojourn time by 2.3 months. Including clinical risk factor data gave a small but significant increment in prediction of referable DR beyond grade (increase in C-statistic of 0.013 in T1D and 0.016 in T2D, both *p* < 0.001). However, using this model to derive personalised intervals did not have substantial workload or sojourn time benefits over stratum-specific intervals. The main limitation is that the results are pertinent only to countries that share broadly similar rates of retinal disease and risk factor distributions to Scotland.

**Conclusions:**

Changing current policies could reduce disparities in ID and achieve substantial reductions in workload within the range of IDs likely to be deemed acceptable. Our tool should facilitate more rational policy setting for screening.

## Author summary

**Why was this study done?:**

- People with diabetes are advised to have their eyes photographed regularly to detect early retinal changes so that they can be referred for preventive treatments for diabetic retinopathy. In some countries, including Scotland, national health service programmes of regular screening have been set up.
- Deciding how often such screening should occur is a complex problem that depends on the individual risk of developing disease but also on system-level issues such as how much screening workload can be delivered, what level of disease progression between screens is deemed acceptable (interval disease risk), whether the system should be designed to ensure this risk is equal for all (equity), and the acceptable time for someone to be in a progressed state before detection (sojourn time).
- Our aim was to use contemporary data from the Scottish screening programme accrued over 10 years to enable policy makers and individuals to make better informed decisions about how often screening should occur and the design of screening programmes. Previous studies were much smaller, used data from earlier decades, when disease rates were higher, or only examined type 1 diabetes (T1D) or type 2 diabetes (T2D), not both.

**What did the researchers do and find?:**

- We took data on retinal photograph screenings from 128,606 screening examinations in people with T1D and 1,384,360 screenings in people with T2D with associated clinical risk factor data collected between 2007 and 2016. We quantified the current rates of disease occurrence and built a predictive model to allow those at more risk to be differentiated from those at lower risk.
- We used the current rate data and the risk model to quantify what the workload, sojourn time, and equity effects would be of different screening interval policies. We created an online tool to enable policy makers to evaluate the effects of different choices about screening intervals.
- The current system used in Scotland and elsewhere achieves low overall risks, but with large differences in risks between individuals.
- The prediction modelling showed that using lots of clinical risk factor data refines the prediction of progression of disease only slightly beyond using just diabetes type and the information contained in the last retinal photograph.

**What do these findings mean?:**

- We show how much the current system could reduce differences in risk by screening some people more often and others less often, and show that can be achieved at the same time as substantially reducing workload.
- The main limitation is that the results are pertinent only to countries that share broadly similar rates of retinal disease and risk factor distributions to Scotland, where the study was done.

## Introduction

Screening people with diabetes using fundus photography with subsequent referral for specialist care of those graded as having sight-threatening diabetic retinopathy (DR) is effective in limiting visual impairment caused by DR [1].

Several countries, including Iceland [2], England [3], and Scotland [4], have introduced national screening programmes. International recommendations for screening intervals are based on the level of DR at previous screenings [5]. In Scotland, individuals are stratified into those with no or mild prior DR, who are screened annually, and those with moderate DR, who are examined every 6 months; those with more severe retinopathy or maculopathy are referred to an ophthalmology clinic [6]. In the US, opportunistic screening is recommended, with screening frequency based on the DR grade of the most recent prior examination [5].

These recommendations, though often not stated explicitly, are based on the assumption that people are to be rescreened when their risk of developing sight-threatening DR reaches a certain threshold. Designing evidence-based screening policies requires a discussion of an acceptable interval disease risk (ID) of referable DR as well as the level to which the risk should be equalised across patients. Current guidelines specify defining screening intervals based on prior DR levels, implying at least some intent to reduce disparities in ID across strata of prior DR. As individual DR risks can vary widely within any stratum of prior DR grade [6,7], personalising screening intervals to equalise IDs at the individual level might also be considered. Such considerations of acceptable ID and equity have important impacts on workload (annual number of screenings required) and sojourn time (length of time a person who develops disease in the interval between screens was in that state before detection). However, these aspects are rarely discussed in designing policies at the moment, as contemporary data on rates of retinopathy progression as well as individual risk factor data for prediction models were not available at the population level until recently. In comparison to previous studies [8–10] on this topic, where the sample sizes ranged from just 1,400 to 11,500 persons and diabetes type was sometimes restricted to type 1 diabetes (T1D) [10] or type 2 diabetes (T2D) [11], we have included data from over 220,000 persons with T2D and 19,000 persons with T1D.

Our goal was to evaluate the workload and sojourn times that result from a range of policy options given current rates of DR progression and to provide national policy makers, guideline committees, and clinicians with an evidence-based interactive tool to aid policy choices (the tool can be accessed at https://diabepi.shinyapps.io/ScreeningIntervals/). To do so, we harnessed a large database capturing DR grade data for 10 years of the national screening programme in Scotland and a national diabetes register with other important clinical risk factor data.

## Methods

### Data sources

This study uses data from the Scottish Diabetic Retinopathy Screening Programme (DRS), which became nationwide in 2007, inviting all patients with diabetes aged ≥12 years for annual screening [6]. DRS screening records were acquired via Scottish Care Information–Diabetes (SCI-Diabetes), a national diabetes register with an estimated coverage of more than 99% of the current diabetes population in Scotland [12]. DRS provided data on the date and outcome of the screening for each patient. SCI-Diabetes provided date of birth, date of diagnosis, type of diabetes, sex, and clinical risk factors. Prior hospital admissions for cardiovascular disease were obtained through linkage to the Scottish Morbidity Record. Date of death was available from the General Register Office for Scotland. Data were available from 1 January 2007 to 31 December 2016.

### Study population

All people with T1D or T2D screened at least once between 2007 and 2016 were included in the dataset. We considered that, in all realistic policies, people with diabetes would be offered an initial screening visit and then 1 more screen after 1 year so as to pick up rapid progressors, and thereafter specification of subsequent intervals would use the results obtained from each patient’s 2 most recent DR screenings. In Scotland the first screening visit is not offered until age 12 years in those diagnosed below this age. Accordingly, for fitting statistical models, the cohort was defined as individuals with at least 3 retinopathy screens, out of which the first 2 had not shown a referable state. Individual observation period began at the day of first screening. Observations were censored at first referable state, death, or the end of the study period. The second screening examination for each individual was designated as the baseline examination. This dataset included 128,606 examinations on 19,070 individuals with T1D and 1,384,360 examinations on 220,276 individuals with T2D; 5,253 individuals with T1D and 17,042 with T2D were excluded because they were in a referable state at their first or second screening.

### Retinopathy grades

DR examinations were carried out using single central 45^○^ field digital photograph, with mydriasis if required [6]. Each eye is graded as 1 of 5 retinopathy (R) grades (R0 through R4) broadly based on the Early Treatment Diabetic Retinopathy Study (ETDRS) scale [13,14] and 1 of 3 maculopathy (M) grades (M0, M1, M2) [14] (see S1 Table for the grading criteria). Any individual graded with R3, R4, or M2 in either eye is referred to an ophthalmology clinic for specialist follow-up. The motivation in referring M2 states is so that the patient can be assessed for the presence of significant macular oedema, which requires 3-dimensional optical coherence tomography (OCT). Lesions on photographs lack specificity for oedema [15,16], and so the majority of those with M2 do not have confirmed oedema and are returned to the screening system. Thus, given the high false-positive rate of referable maculopathy states (M2), referable DR in this study is defined as being graded R3, R4, or M2 and not being subsequently rescreened.

### Clinical risk factors

Clinical measures from routine healthcare available in SCI-Diabetes included age, sex, diabetes duration, BMI, HbA1c, systolic and diastolic blood pressure, blood lipids, estimated glomerular filtration rate, ever/never smoking status, and statin and anti-hypertensive drug use. Measures of clinical variables were taken from the measure nearest to and up to 730 days prior to each patient’s screening episode. Where clinical risk measures were unavailable during this time, values were imputed, using the *mi* package for *R* [17].

### Statistical analysis

The key steps involved were to (i) quantify rates of transition to referable DR, (ii) build risk prediction models for referable DR separately for T1D and T2D, and (iii) use the risk estimates from these models to quantify the expected workload and sojourn time from either setting various IDs to be equal at stratum-specific or individual levels, or choosing intervals at set times.

Technical details are given in S1 Text. Generalised linear models with complementary log-log link function to allow for interval censoring were used to model the transition to referable DR from the second screening episode. Several models, including previously published ones, were constructed with predefined sets of covariates. The models were fitted to a training dataset, containing a randomly selected 70% of individuals, and their predictive performance was evaluated on a test dataset, containing the remaining 30%. Predictive performance was evaluated using the C-statistic, and the log likelihood test was used to evaluate the strength of the evidence that the model improved prediction compared with a base model containing the penultimate 2 DR grades. We modelled the hazard function as constant over time, equivalent to a Poisson arrival process and to an exponential distribution of times to failure. This is adequate given the short intervals modelled in this study. Of note, fitting a Weibull model that allows for the hazard function to vary over time did not improve the models’ predictive performance.

We considered first the fit of the models and then chose the model that was most sparse among those with the best fit. The chosen model was then used on the data to calculate for each patient the hazard rate of transitioning to referable DR with different time intervals between screenings. These hazard rates for each individual were used to generate the intervals that would be required under various settings to achieve a specific ID and setting the ID to be the same (i) for each individual or (ii) across strata of prior DR. For a personalised optimal screening schedule, the interval for each individual was calculated such that the probability of transition to referable DR equated to the target ID. For stratum-level intervals, we computed the interval that would be required such that the average probability of transition to referable DR across all patients in each stratum by the end of that interval equated to the chosen ID.

The annual workload and average sojourn time for a range of policy options in terms of varying overall ID and achieving either stratum-level or personal-level equity of IDs were then assessed and compared to the current policy in Scotland.

An outline analysis plan of the key steps of calculating current referable retinopathy rates, building a prediction model, and using the model to determine the estimated number of screenings required maintain the ID below a given threshold was set in advance (S1 Statistical Analysis Plan), but many aspects of this plan evolved during the analysis. For example, we decided that a generalised linear model with complementary log-log link function to allow for interval censoring was more appropriate than the Poisson model we initially specified. We had initially considered examining just 1 model, evaluating covariates reported in the literature as predicting retinopathy risk and using backward elimination, but later realised we should explore a wider range of models, including models previously reported. We had not initially considered sojourn time as an important impact to be assessed and added this later. This study is reported as per the Strengthening the Reporting of Observational Studies in Epidemiology (STROBE) guideline (S2 Table). This study was approved by the Public Benefit and Privacy Panel for Health and Social Care (https://www.informationgovernance.scot.nhs.uk/pbpphsc/; application reference 1617–0147) and by the Scotland A Research Ethics Committee (Ref. 11/AL/0225).

## Results

At baseline examination, the average age and diabetes duration of the T1D patients (*n* = 19,070, 45.8% female) were 36.6 (SD 17.3) and 13.2 (SD 12.3) years, respectively. For the 220,276 T2D patients (43.9% female), age and diabetes duration were 62.9 (SD 12.2) and 4.1 (SD 5.4) years, respectively. Further baseline characteristics are shown in Table 1.

**Table 1:** Clinical measures at baseline in patients with type 1 and type 2 diabetes who did versus did not develop referable diabetic retinopathy over the study period.

Characteristic	Type 1 diabetes			Type 2 diabetes		
	Not referable	Referable	*p*-Value*	Not referable	Referable	*p*-Value*
Age at diagnosis (years)	26.1 (15.1) 24.5 [13.9; 35.6]	23.6 (13.7) 22.3 [12.5, 32.9]	<0.001	57.6 (11.8) 58.0 [49.8; 65.9]	51.8 (12.4) 51.2 [43.3; 60.0]	<0.001
Female (%)	47.0	44.1	<0.001	43.8	42.3	<0.001
Diabetes duration at screening (years)	21.4 (12.8) 19.4 [11.5; 29.8]	22.5 (10.3) 20.3 [14.9; 27.6]	<0.001	9.3 (6.7) 8.3 [4.4; 12.8]	13.5 (6.6) 12.9 [9.0; 17.0]	<0.001
BMI (kg/m^2^)	26.7 (4.6) 26.1 [26.6; 29.0]	27.3 (4.4) 26.8 [24.1; 30.0]	<0.001	31.9 (6.2) 30.9 [27.6; 35.1]	32.0 (6.3) 30.9 [27.5; 35.3]	0.376
Height (m)	1.7 (0.1) 1.7 [1.6; 1.8]	1.7 (0.1) 1.7 [1.6; 1.8]	<0.001	1.7 (0.1) 1.7 [1.6; 1.8]	1.7 (0.1) 1.68 [1.6; 1.8]	0.053
HbA1c (mmol/mol)	68 (15.7) 66.0 [57; 76]	75.9 (16.2) 74.0 [65.0; 85.8]	<0.001	57.5 (15.6) 54.0 [48.0; 64.0]	68.0 (19.1) 64.0 [54.0; 79.0]	<0.001
Systolic blood pressure (mm Hg)	129.3 (16.1) 130.0 [119; 140]	130.0 (16.0) 130.0 [120.0; 140.0]	0.031	134.7 (15.7) 134.0 [125.0; 142.0]	136.8 (16.3) 136.0 [126.0; 145.0]	<0.001
Diastolic blood pressure (mm Hg)	74.5 (9.9) 75.0 [68.0; 80.0]	75.3 (9.7) 75.0 [70.0; 81.0]	0.817	76.2 (9.7) 77.0 [70.0; 82.0]	76.9 (10.1) 78.0 [70.0; 82.0]	<0.001
Total cholesterol (mmol/l)	4.6 (0.9) 4.5 [4.0; 5.1]	4.8 (1.0) 4.6 [4.1; 5.3]	<0.001	4.3 (1.0) 4.2 [3.7; 4.8]	4.3 (1.0) 4.2 [3.7; 4.9]	<0.001
HDL cholesterol (mmol/l)	1.6 (0.5) 1.5 [1.3; 1.8]	1.6 (0.4) 1.5 [1.3; 1.8]	0.085	1.2 (0.4) 1.2 [1.0; 1.4]	1.2 (0.4) 1.2 [1.0; 1.4]	<0.001
LDL cholesterol (mmol/l)	2.4 (0.9) 2.4 [1.9; 2.9]	2.6 (0.8) 2.4 [2.0; 3.0]	<0.001	2.3 (0.9) 2.1 [1.6; 2.7]	2.2 (0.9) 2.1 [1.6; 2.7]	0.004
Triglycerides (mmol/l)	1.3 (1.6) 1.1 [0.7; 1.5]	1.4 (1.0) 1.1 [0.8; 1.8]	<0.001	2 (1.2) 1.8 [1.3; 2.5]	2.1 (1.7) 1.8 [1.3; 2.6]	<0.001
eGFR (ml/min/1.73 m^2^)	91.8 (20.8) 93.0 [78.0; 106.6]	93.9 (20.7) 95.9 [80.3; 108.8]	<0.001	75.2 (19.9) 75.6 [61.4; 90.1]	77.4 (21.3) 78.9 [62.4; 93.7]	<0.001
Anti-hypertensive drugs (%)	23.2	24.8	0.003	70.1	66.4	<0.001
Statins (%)	29.3	32.8	<0.001	68.2	68.8	0.042
Ever smoker (%)	60.7	66.2	<0.001	72.1	69.8	<0.001
Past CVD event (%)	5.2	5.6	0.162	22.2	20.7	<0.001
Normal vision (%)	99.2	99.5	0.001	98.6	98.6	0.581

For continuous variables, the first row gives mean (SD), and the second row gives median [IQR]. For categorical variables, percentage is shown.

*p-Values of univariate tests of differences between patients who transitioned to referable diabetic retinopathy and those who did not over the study period.

CVD, cardiovascular disease; eGFR, estimated glomerular filtration rate; HDL, high-density lipoprotein; LDL, low-density lipoprotein.

### IDs for current policy

Under the currently implemented screening schedule, the estimated overall average risk of developing referable DR after 1 year of screening and 2 initial non-referable screenings was much greater for T1D (2.4%) than for T2D (0.6%). The current schedule yields also very different IDs across prior DR strata: 0.6% per year, 3.6% per year, and 7.1% per 6 months in those with no, mild, and moderate retinopathy, respectively, in T1D, and respective IDs of 0.2%, 1.7%, and 6.7% in those with T2D. Estimates of the predicted probability of transitioning to referable DR across various time points by DR stratum are shown in Table 2.

**Table 2:** Unadjusted predicted probability (in percent) of transition to referable DR at given time intervals, stratified by previous grade (no, mild, or moderate DR).

Time interval	Type 1 diabetes			Type 2 diabetes		
	No DR to referable	Mild to referable	Moderate to referable	No DR to referable	Mild to referable	Moderate to referable
1 month	0.1	0.3	1.3	<0.1	0.1	1.2
2 month	0.1	0.6	2.5	<0.1	0.3	2.4
3 month	0.2	0.9	3.7	0.1	0.4	3.5
6 month	0.3	1.8	7.1	0.1	0.9	6.7
9 month	0.5	2.7	10.2	0.2	1.3	9.7
1 year	0.6	3.6	13.2	0.2	1.7	12.5
2 year	1.2	7.0	23.8	0.4	3.4	22.0
3 year	1.8	10.2	32.6	0.6	5.0	29.9
4 year	2.4	13.3	40.1	0.8	6.6	36.5
5 year	3.0	16.2	46.5	1.0	8.1	42.2

Rates of transition to referable DR predicted at different time intervals using model including penultimate 2 DR grades and patient’s age at grading, sex, diabetes duration, HbA1c, and total cholesterol (Model 5 in S3 Table).

DR, diabetic retinopathy.

### Maximising prediction of transition to referable retinopathy

The model containing prior grade and demographics (age, sex, and diabetes duration) and constrained to select only 2 additional risk factors (HbA1c and plasma total cholesterol—Model 5 in S3 Table) performed as well on the data as less sparse models and was therefore used to derive risk-based screening intervals. Using this model, the C-statistic increased modestly from 0.757 (95% CI 0.738; 0.775) to 0.771 (0.753; 0.790) for T1D and from 0.778 (0.767; 0.788) to 0.794 (0.784; 0.805) for T2D compared to a base model of the penultimate 2 DR grades alone. The difference in test log likelihoods for the model versus the base model was significant at *p* < 0.001.

### Comparing screening schedules

**Fig 1:**
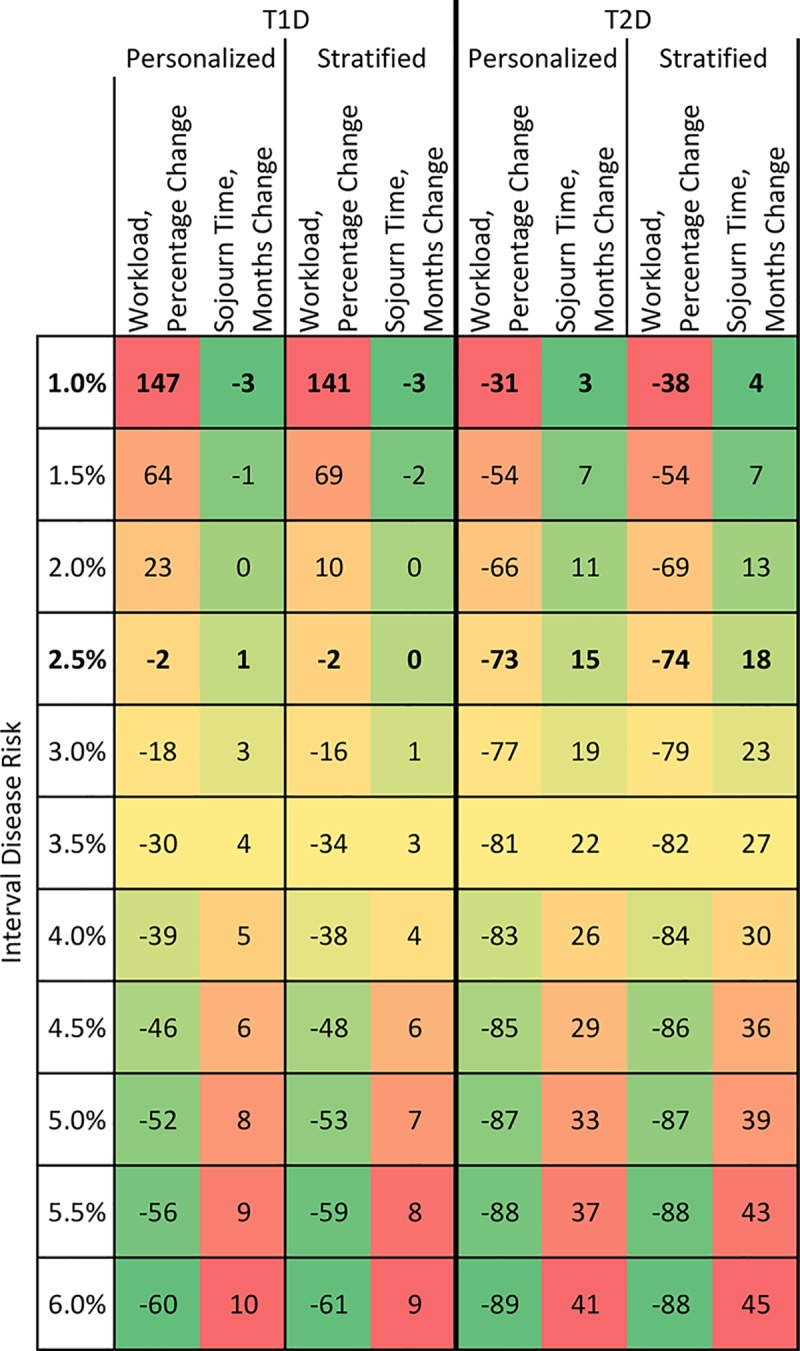
Comparison of screening schedules in terms of changes in number of required screenings and sojourn time relative to the currently implemented policy. Heatmap showing policy implications for a number of personalised and stratified schedules for patients with type 1 diabetes (T1D) and type 2 diabetes (T2D) in terms of changes to workload (percentage change) and sojourn time (change in months) compared to the current policy. Screening schedules are varied by accepted interval disease risks (*y*-axis) and personalised or stratified setting (*x*-axis). Red shades show large increases and green shades show large decreases in workload or sojourn time compared to the current screening schedule, with yellow shades showing intermediate levels of change. For example, a personalised screening schedule for patients with T1D with an accepted interval disease risk of 4.0% would reduce overall workload by 39% and sojourn time by 5 months compared to the currently implemented policy.

Table 3 shows the required screening intervals and implications for workload and sojourn time of modifying the current screening schedule to achieve the same overall ID of referable DR as the current policy (2.4%/0.6% for T1D/T2D) but specifying that the ID at point of screening to be equal (i) at individual level or (ii) across prior DR strata. Under both of these options, intervals become very long for patients with no prior disease (47 months in T1D for example) and very short for those with moderate disease (2 months in T1D). Equalising IDs across strata at the current overall ID had little impact on workload or sojourn time in either type of diabetes. The heatmap in Fig 1 summarises the impact, with respect to the current policy, of varying the acceptable ID threshold under policies aiming to equalise risks across strata or individuals. For example, a policy of setting the ID for T2D to be 2.4% overall (similar to the current ID for T1D) and equalising within strata would substantially reduce the workload, by 73%. Screening intervals would be 140, 17, and 2 months for those with no, mild, and moderate DR, respectively. The recommended intervals under other varying ID thresholds can be evaluated quickly using the online tool. A general observation from Fig 1 is that personalised schedules, whilst by definition achieving equity, do not offer benefits in workload or sojourn times over the range of IDs considered.

**Table 3:** Workload and sojourn time effects of setting screening intervals to equalise stratum-specific or personal risks of referable disease at the current interval disease risk.

Prior DR grade	Type 1 diabetes					Type 2 diabetes				
	Interval in months, median (IQR)	Interval disease risk percent	Number of required screenings	Percent workload change from current policy	Average sojourn time in months (% change)	Interval in months, median (IQR)	Interval disease risk percent	Number of required screenings	Percent workload change from current policy	Average sojourn time in months (% change)
**Currently implemented policy**										
None	12	0.6	10,363			12	0.2	174,159		
Mild	12	3.6	7,813			12	1.7	43,887		
Moderate	6	7.1	1,787			6	6.7	4,460		
Total		2.4	19,963	—	5.9 (0.0)		0.6	222,506	—	6.1 (0.0)
**Personalised schedule, current overall interval disease risk**										
None	61 (40–87)	2.4	3,027			42 (31–51)	0.6	65,091		
Mild	9 (6–18)	2.4	11,883			6 (3–10)	0.6	125,683		
Moderate	2 (2–4)	2.4	5,505			1 (0–1)	0.6	65,639		
Total		2.4	20,415	2.3	6.8 (14.4)		0.6	256,413	15.2	5.2 (−15.3)
**Stratified schedule, current overall interval disease risk in each stratum**										
None	47	2.4	2,646			35	0.6	59,712		
Mild	8	2.4	11,720			4	0.6	131,661		
Moderate	2	2.4	5,358			1	0.6	26,760		
Total		2.4	19,724	−1.2	5.8 (−2.5)		0.6	218,133	−2.0	6.0 (−2.2)

For personalised and stratified schedule, screening intervals were derived by estimating the time to rescreen when set interval disease risk is reached, using Model 5 in S3 Table. From this, the annual number of screenings required was estimated and change from current policy computed.

Prior DR grade: patient’s DR grade at last screening.

Interval in months: median (IQR) of predicted screening interval for personalised schedule; predicted screening interval for stratified schedule.

Interval disease risk percent: predicted interval disease risk in percent of referable DR for currently implemented policy; set interval disease risk in percent for personalised and stratified schedules. For stratified schedule, average interval disease risk is across all patients within prior DR strata. For personalised schedule, interval disease risk is personalised risk for each patient.

Number of required screenings: number of screenings required annually based on the current diabetes population in the last year of the programme and the predicted interval for individual patients or strata.

Percent workload change from current policy: percentage change in number of screenings required annually in comparison to number of screenings required annually for currently implemented policy.

Average sojourn time in months (% change): average time (in months) elapsed between onset of referable DR and detection during next screening, with percentage change from currently implemented policy in parentheses.

DR, diabetic retinopathy.

Table 4 illustrates the workload, sojourn times, and effective stratum-specific ID and degree of equity achieved with some sample schedules that specify intervals rather than aiming to achieve a given ID. The reader can evaluate other schedules using the interactive tool. It is clear from these data that without changing to diabetes-type-specific schedules, large disparities in IDs between T1D and T2D will remain. For example, as shown using the interactive tool, changing to intervals of 12, 9, and 3 months in T1D and 24, 9, and 3 months in T2D for no, mild, and moderate DR strata, respectively, would substantially reduce inequity across strata and between diabetes types whilst reducing workload by 26% (T1D and T2D combined) and would increase average sojourn time by just 2.3 months.

**Table 4:** Screening schedules using a range of potential different screening intervals.

Prior DR grade	Type 1 diabetes				Type 2 diabetes			
	Interval in months	Interval disease risk percent	Percent workload change from current policy	Average sojourn time change in months	Interval months	Interval disease risk percent	Percent workload change from current policy	Average sojourn time change in months
**12/9/3 months (T1D) and 24/9/3 months (T2D) screening schedule**								
None	12	0.6			24	0.4		
Mild	9	2.7			9	1.3		
Moderate	3	3.7			3	3.5		
Total		2.0	22.0	−1.1		0.9	−30.6	2.8
**36/9/6 months (T1D) and 36/18/6 months (T2D) screening schedule**								
None	36	1.8			36	0.6		
Mild	9	2.7			18	2.6		
Moderate	6	7.1			6	6.7		
Total		3.0	−21.6	1.8		1.5	−58.8	9.4
**24/12/6 months (T1D) and 24/12/6 months (T2D) screening schedule**								
None	24	1.2			24	0.4		
Mild	12	3.6			12	1.7		
Moderate	6	7.1			6	6.7		
Total		3.2	−25.9	2.2		1	−39.1	4.1

For each of the schedules, the intervals in months were set, and Model 5 in S3 Table was used to derive the average expected interval disease risk given each of the intervals. Intervals and numbers of patients in each stratum were used to derive expected workload and compute change in workload compared to current policy.

Prior DR grade: patient’s DR grade at last screening.

Interval in months: set screening interval per stratum.

Interval disease risk percent: expected interval disease risk of referable DR (in percent) for given interval.

Percent workload change from current policy: percentage change in number of screenings required annually in comparison to screenings required annually for currently implemented policy.

Average sojourn time change in months: change (in months) in average sojourn time in comparison to that of currently implemented policy.

DR, diabetic retinopathy; T1D, type 1 diabetes; T2D, type 2 diabetes.

## Discussion

### Key findings

By using a large dataset from a national DR screening programme, we have been able to establish some key points that we hope will help retinopathy screening guideline committees and policy makers in many countries. First, we found that currently implemented schedules, both in Scotland and elsewhere, result in large disparities in ID between T1D and T2D, and between those with differing prior DR grades: risks of T2D with no prior DR are 0.5%, compared to 15.4% for people with T1D and prior moderate DR. These disparities are of a magnitude likely to warrant change in current guidelines and screening schedules. Second, across the range of IDs we considered, annual screening for low-risk patients with no prior DR is unnecessary, especially for T2D but, it could be argued, also for T1D. More frequent examination or even immediate referral of those with moderate DR should be considered, but this will depend on the ID considered acceptable.

Third, we found that the implications of varying ID criteria and equity considerations for workload and sojourn times are not intuitive; this is because the impact depends on the relative frequency of the strata, the range of individual risk, and variance in risk factors, as well as the ID thresholds and equity criteria being set. Our tool facilitates formal estimation of these impacts.

Fourth, we find that to make stratum-specific IDs completely equal would result in very long and short intervals for those with no and moderate prior disease, respectively, across the range of IDs 1%–6% that we show. We doubt that patients or clinicians would find intervals of over 3 years or under 3 months acceptable, but this should be explicitly considered. Fifth, the data suggest that whilst seeking perfect equity is likely infeasible, substantial reduction in the disparity in ID by diabetes type and prior DR stratum can be achieved at the same time as achieving substantial reductions in workload across the range of IDs to be under consideration by policy makers.

Rational design of DR screening schedules requires consideration of acceptable IDs, the extent to which equity across broad groups such as type of diabetes or prior disease grade or between individuals is desirable, sojourn time, and whether there are upper or lower boundaries on acceptable screening frequency. These concepts are not widely discussed or made explicit in current clinical guidelines or national programmes, mainly because the necessary underpinning data on contemporary disease rates have not been available. We hope our data and approach will help to inform guideline development in future.

Although personalised intervals have been advocated in particular in terms of equity, the benefits of this approach in terms of workload and sojourn time have been unclear. To our surprise, our analysis did not make a clear-cut case for using extensive clinical risk factor data to personalise risk intervals. We had in fact set out to develop such a personalised tool, as others have attempted, for instance the Retina Risk tool (https://retinarisk.com), developed by Einar Stefansson and colleagues, thinking that personalisation would likely yield considerable benefits. We found that risk factor data did significantly improve prediction of the referable state on top of using prior grades, but the magnitude of the increment was small and most of it could be captured using a fairly sparse set of variables. This does not mean that these risk factors are not important in retinopathy; more likely is that their contribution is already captured in the grade information. To get a sense of the difference in just assigning the same interval to everyone with a given grade versus using the risk factor model to assign an interval, the reader can compare the data in the second and third schedules of Table 3; for T1D, if one sets a stratum-specific interval such that, overall, 2.4% of those with no prior DR have DR at the next screening, then all would be assigned an interval of 47 months, with the risk for individuals varying around an average of 2.4%. Using the model to set intervals such that everyone has an ID of 2.4%, the median interval for those with no prior DR would be 61 months (IQR 40–87 months). These data show that although the personalised approach would achieve perfect equity of ID, a practical issue is that the extremes of the predicted intervals are such that they would likely be deemed unacceptable to implement anyway. The reader can explore the range of predicted personalised intervals across other ID thresholds using the online tool (https://diabepi.shinyapps.io/ScreeningIntervals/). What our analysis also shows is that if inequity of risk is the main concern, then the first priority should be to set intervals to reduce the inequity between T1D and T2D and between differing prior grade strata. As shown in the heatmap in Fig 1, across a range of acceptable IDs there is little to argue for a personalised approach in terms of workload.

### Strengths and weaknesses

A particular strength of this study is that it is based on a total population that is representative of a nationwide diabetes population of more than 200,000 people, unlike previous studies that contain only 11,806 people [9] or fewer. It includes DR screening results from both T1D and T2D patients, compared to other studies that included only one diabetes type [10,11]. Furthermore, it uses results of DR screening over a 10-year period in a contemporaneous real-world setting. Another strength is the rich risk factor data that we had available, which enabled us to evaluate the increment in prediction from considering such data. We note our prediction models outperformed published models [8,10] when fitted to the Scottish population. This is likely to be in part because we used a more detailed measure of 2 previous DR grades, rather than just 1 previous DR grade.

A limitation of our analysis is that it is necessarily based on assuming that patients perfectly adhere to the proposed schedules, but it is likely that in the real world adherence will be imperfect, so that effective outcomes will differ slightly. A limitation of modelling referable DR as a Poisson arrival process is that it does not allow for observer variability in grading of referable disease or for possible regression of this state between examinations. A hidden Markov model, implemented for instance in the *msm* package in *R*, can allow for this and might achieve slightly more precise estimation of rates. We have used this approach in the past on smaller datasets, and it was used for the DCCT/EDIC Research Group [10] analysis on a smaller dataset, but it has computational limitations especially when modelling the effects of many covariates on the hazard function.

Vision-threatening DR (VTDR) risk is also important for policy makers, guideline committees, and clinicians. VTDR is a less common, more specific subset of the referable state that our system defines (approximating having R4 or macular oedema). However, it requires 3D OCT such that the macular oedema component can be definitively diagnosed. From the results of the 2D photograph screening in the screening programme, it is not possible to assign which patient has VTDR, only which has the macular changes referred to as M2 that are a 2D proxy for risk of oedema. Therefore, strictly speaking, we cannot model VTDR. That said, since VTDR is less common than the referable state, modelling it alone would likely lead to longer, not shorter, screening intervals being recommended (since ID would be lower). As such, the intervals recommended are likely to be conservative with respect to VTDR. For this not to be the case, very different risk factors with very different weights and a very different relationship of prior grade to VTDR incidence than to referable disease incidence would have to pertain, and this is of course worth directly testing as data become available. Of note, the current plan in Scotland is to take OCT into the screening system over the next few years. When that happens, and as those data accrue, we will be able to analyse VTDR as a specific outcome.

An important issue for all studies of this question is the extent to which conclusions can be generalised from one setting to another. Our data reflect a total population and are not restricted, for example, by the more specific entry criteria of a clinical trial. They are also contemporaneous. The results will be broadly applicable to countries with retinopathy rates similar to ours and with similar risk factor distributions. Glycaemic control is slightly worse in Scotland compared to other Western countries [18]: To the extent that this impacts on disease risk, it will mean that the predicted screening intervals will be slightly longer than for countries with lower rates. However, to confirm the intervals predicted in this study, external validation using other countries’ data will be required. It should be noted that the current screening system in Scotland and elsewhere has been designed to ensure referral of those likely to benefit from currently approved treatments. If the evidence base for treatments were to change, resulting in recommendations for interventions at an earlier stage of retinopathy, this would require a redesign of the programme and a reanalysis of the then appropriate screening intervals.

In conclusion, we have provided a framework and tool for policy makers to more formally consider the intervals needed for retinopathy screening in diabetes. Compared to current policies, we show that reductions in workload, with consequent substantial savings to healthcare budgets, and reductions in disparity of risks between groups can be achieved.

## Supporting information

S1 Statistical Analysis Plan (PDF)

S1 Table Retinopathy and maculopathy grades and retinopathy severity definitions. (DOCX)

S2 Table STROBE checklist. (DOCX)

S3 Table Comparison of predictive models for type 1 and type 2 diabetes. (DOCX)

S1 Text Details of methods. (DOCX)

## Abbreviations

DR: diabetic retinopathy
DRS: Scottish Diabetic Retinopathy Screening Programme
ID: interval disease risk
OCT: optical coherence tomography
SCI-Diabetes: Scottish Care Information–Diabetes
T1D: type 1 diabetes
T2D: type 2 diabetes
VTDR: vision-threatening diabetic retinopathy
