# Supplementary material for: Use of personalised risk-based screening schedules to optimise workload and sojourn time in screening programmes for diabetic retinopathy: A retrospective cohort study
Source: PLoS Med. 2019 Oct 17;16(10):e1002945. doi: 10.1371/journal.pmed.1002945 (PMC6797087; doi:10.1371/journal.pmed.1002945)
Supplement: S1 Statistical Analysis Plan — (PDF) [file pmed.1002945.s001.pdf]

CSO Retinopathy Screening Project statistical Analysis Plan

V1

13.01.17

Aims

We aim to develop risk prediction tools that can be used by retinopathy screening services to define the optimal interval to next screening in the national screening programme taking into consideration past screening records and other cvariates available in SCI-DM

Objectives

To further this aim we will

I)first calculate the currently observed age and sex and diabetes type specific rates of first referable retinopathy in the screening programme and describe these by calendar year

2) Build a predictive model and validate its prediction performance of time to first referable disease

3) Extend that nodel to utilise a hidden markov model derived measure of risk and assess whether this improves predictibe performance

4) compare the estimated number of screenings required to maintain interval disease rate below a given threshold using the maximally predictive model from this process with a much simpler decision algorithm such as the current programme and a programme that would screen all type 1s annually and all types 2s biannually.

The models will be developed for type 1 and type 2 separately

Data sources

Scotland:

Anonymised data from a national clinical database of all patients with a READ code in their clinical record for diabetes mellitus (The Scottish Care Information – Diabetes Collaboration -SCI-DC/SCI-DM) system or more recently SCI-Diabetes). The assignation of such a diagnostic code triggers entry to the database. The most recently available data is the SCI-DM extract of May 2014 but this is currently being updated with data to late 2016.

SCI-DC data contain extensive info including all clinical measurements lab tests urine tests and issued prescriptions as well as annual retinopathy screening data. The data are linked anonymously to hospital admissions data (Scottish Morbidity Record SMR-01), held by the Information Services Division of the National Health Service (NHS) back to 1981, and death data held by the General Register Office for Scotland, using the CHI healthcare number with probabilistic linkage (mal-linkage rate <3%) [1].

Inclusion criteria, entry and exit times

The study will focus on the diabetes populaiton aged 12 years and upwards since this is the group eligible for screening in national policy.

Type 1 diabetes : is defined by an algorithm that starts with the clinical assignation of type 1 in the clinical record but then excludes anyone with evidence of type 2 based on extensive oral prescription drug use or more than a year from diagnosis to insulin.

Type 2 diabetes: is defined by the clinical assignation of type 2 unless threis evidence to contradict this.

D*ate of diagnosis:* this is taken from SCI-DM – in a small % of cases date of diagnosis is not known and earliest date known to have diabetes has to be used instead.

Study start date: 01/01/2007

Study end date: 01/06/2016

Patient entry date is defined as the latest of study start date, the date of diabetes diagnosis, and the date first evaluable for events (as defined below). Note that for the modelling purposes patient entry date will be further refined to be S2 day +1.

Patient exit date for a patient is the earliest of

i)study end date,

ii) the date of death,

iii) the date last evaluable for events (as defined below)

iv) or the date of the first event of interest.

Those who exit before an event or study end date are considered to be censored.

Date first evaluable for events

The dates from which individual patients are first considered evaluable for events are estimated from the dates of when either clinical measures (BMI, HbA1C and blood pressure [BP]) or drug prescription data are first available for that person , since these records confirm that a patient was in Scotland at the time and can therefore be considered evaluable for events.

Date last evaluable for events

The date last observable for events is defined as the earliest of

i) the date last observable for routine data or drug data (i.e. exit from the country) whilst not under admission to hospital plus an additional 183 days
ii) death, or 01/06/2016; Sensitivity analyses will evaluate the impact of this 183 day period which is in place so as not to censor people too early who simply haven't had any contact with clinical services during the period but who are still under observation.
iii) Date of suspension from screening for non ey disease related reason. Such patients can re-enter once the suspension has been lifted as evidenced by a further screening event

Endpoints

The primary endpoint will be referable retinopathy

Secondary endpoints are referable maculopathy alone and refereable retinopathy without maculopathy

Baseline Covariates considered for inclusion in referable retinopathy prediction model

The covariates to be considered for inclusion into the model are those reported in the literature as predicting retinopathy risk OR have been included in previous risk models.

Time updated covariates will be sued in the analyses being updated wrt each screening date

- Sex coded
- Ethnicity
- Current age
- Age at diagnosis
- Pt entry date
- The Scottish Index of Multiple Deprivation (SIMD) quintile- this is a residence based measure of socioeconomic status .

- BMI (kg/m2)
- Height, m
- HbA1c mmol/mol (To reduce potential for reverse causation, HbA1c values are to be lagged by at least one year. Start by considering running mean of readings taken between 1 and 3 years prior to baseline.)
- eGFR
- Albuminuria status (1: Normal 2: Micro 3: Macro)

- Systolic blood pressure, mmHg
- Diastolic blood pressure, mmHg
- Treated hypertension (based on prescription of any blood pressure lowering drugs)

- Total cholesterol, mmol/L
- Log (total/HDL) cholesterol
- Also consider non-HDL cholesterol, HDL-c and LDL-c as a set of lipid variables, choosing the combination giving the best model fit?
- Treated elevated cholesterol - ie on Statin therapy or other lipid lowering drugs (No LLT, statins, other LLT)

- Smoking status (categorised as never smoked, ever-smoked, current smoker- we do not think our pack year data are reliable enough to use pack years)

- Screening status at last screening available
    - We will also constrct a weighted average of screening scroes from all preceding episodes

Statistical methods

Data preparation

Missing covariate data

Covariates at a given time *t* will be defined by the status at the most recent assessment available prior to that screening date. If the covariate is not available in a look back up to a max of two years prior to that screening date if should bedeclared as missing.

Covariates missing within this time horizon of patient entry date in more than 40% of participants will be excluded. Where the missingness is < 40% multiple imputation (see statistical methods section) will be used to assign a value based on other variables observed for the patient as well as surrounding values of that particular variable.

Covariate terms will also be set up for age x covariate and sex x covariate interactions for each covariate.

"MI" R package will be used for imputation. 10 iterations with 2 chains will be used, with seed = 1986. No consideration of event status is made in the imputation.

Data formatting

The data are split longitudinally from a patient's entry date to their exit or censor date using time-intervals efined by screening episodes. Ie if a patient has had 5 scrrening episodes after entry (so s1 s2 then 5 after that) this would mean that they have 5 rows in the dataset. Covariates get updated at each screening episode.

Event dates

The date of the event will be taken as the date of screening on which referable retinopathy was detected. Each person time interval has a binary flag for whether an event occurred in a person-time interval.

We also have a censor date (which is the earliest of death/event/study end date/end of observability). If a person is censored and his event flag is 1, then that means he was censored because of the event. If at the person's last time-interval the event flag is 0, then he was censored for one of the other reasons (i.e. akin to a SURV object in R for Cox regression).

Initial tabulations

For each continuous covariate being considered :

- Summarise its distribution as mean median sd iqr and range and plot its distribution (kdensity or histogram) plot
- Tabulate the frequency of all categorical variables

Events:

- Show the number (%) and event rate of incident events during follow up By AGE and SEX and diabetes type
- By Sex strata and for broad age bands and all ages combined show the age standardised event rate for each of the event of interest standardised to the 2013 European Standard Population

Then with any refereable retinopathy as the event of interest :

- Summarise the distribution of follow up time range of person entry and exit dates
- Summarise the distribution of covariates at baseline by subsequent event status

- In the same table show the p value and the beta regression coefficient for the current age - sex -and diabetes duration -adjusted associations of all variables with the outcome

Model construction

Assuming any referable retinopathy as event of interest initially:

a. <u>Poisson regression</u>

We will use Poisson regression model with backward elimination but we may use a different link function to deal with the interval censoring - In addition we will evaluate the use of non-parametric methods for interval-censored survival analysis in place of the interval regression models.

All model fitting will be done on 70% of the data (selected randomly by patient, maintaining ratio of events), and the remaining 30% will be used for internal validation. We will randomly assign the individuals in the dataset into these two partitions.

We include current age, sex, and diabetes duration as our base set of covariates in our initial model and will then use backward elimination to drop variables from the model, using a reduction in AIC of < 2 units as the selection/stopping criterion.

We will initially include a simple covariate of screening status at last screening as a covariate

We will then extend this to be a weighted score of all prior screenings weighted by how recent the measure is

The model will contain an offset term for the log(length of interval).

We will also select across age and sex interaction terms for each covariate.

To be agreed : should we include any any shrinkage/ penalizing?

Assumption about linearity of effects of covariates :

We will use backward elimination with multivariable fractional polynomials

b. <u>Two-step analysis with multi-state modelling & survival analysis:</u>

We will use hidden Markov models to model the DRS data. The advantages to this approach are that it allows us to model the risk for transition incorporating all available data on prior screening even though individuals have variable number of examinations at differing intervals. It also allows us to model grading misclassification as it separates the 'true but unseen grade' from the 'observed grade'. We will fit separate models by diabetes type and the models will be adjusted for age, sex and diabetes duration. As it is not feasible to extend the multi-state modelling approach to a more complex model that includes many clinical covariates we will "plug in" the risk score computed from the hidden Markov model into the fuller model that includes all available clinical covariates as escribed above.

One-step survival analysis: We will evaluate an alternative approach of not using the hidden Markov model but instead fitting separate models for individuals with 0, 1, 2 or more previous examinations. This is possible as we have a large dataset so that we will have an adequate sample size to learn a predictive model within each of these strata.

Non-parametric methods: We shall also evaluate non-parametric methods for interval-censored survival analysis. These kernel-based methods, which make predictions based on a function that evaluates the similarity between pairs of observations, generally outperform simple regression models for prediction though the predictions are not readily explainable.

Performance evaluation

The performance of the final model will then be reported from the test dataset.

Discrimination will be assessed using a C-statistic (area under the receiver operating characteristic [AUROC]). We will calculate the AUROC within age and sex strata and then take a weighted average of these to define the final AUROC .

Calibration will be summarized using a calibration plot, calibration slope, and Hosmer-Lemeshow test.

We will also report out the net reclassification indices and the IDI. However note that we consider the AUROC to be the most valid of these. Integrated Discrimination Index (IDI). The IDI =(IS_{new} −IS_{old})−(IP_{new} −IP_{old}) where IS is the integral of the sensitivity across all possible cut-off values for the new (IS_{new}) and old models (IS_{old}) and IP the corresponding integral of 1-specificity with the new model reflecting the model with added clinical covariates and the old model being based only on the DRS screening data. These metrics will allow us to assess how well these models improve prediction of risk for transition to referable eye disease.

In the test set we will compare the predictive performance of the prediction model with this hidden markov derived measure wih the simpler indices of past screening results as described under a. above

Utility assessment

To be expanded: We will compare the estimated number of screenings required to maintain interval disease rate below a given threshold using the maximally predictive model from the above process with a much simpler decision algorithm such as i) the current annual screening for all programme and ii) a programme that would screen all type 1s annually and all types 2s biannually.

Since there are many ways in which policy formulations might utilise prediction risk. We will produce some examples; for instance we can show what the impact on number of screens per annum based on individualised screening intervals set by a series of thresholds of risk (e.g. 1% or 2.5% or 3%). We can also calculate the number of people who will potentially have a delay of >3 months in detection of referable disease and calculate the median 'delay' for each scenario (i.e. the interval between when they would have been screened based on current screening scheme and when they would be screened under the new scheme). The aim is that the models will be able to evaluate a wide range of potential screening policies to provide interactive feedback to policy makers.

Adherence to standards :

This protocol has been developed to be compliant with the TRIPOD statement
