# Supplementary material for: Use of personalised risk-based screening schedules to optimise workload and sojourn time in screening programmes for diabetic retinopathy: A retrospective cohort study
Source: PLoS Med. 2019 Oct 17;16(10):e1002945. doi: 10.1371/journal.pmed.1002945 (PMC6797087; doi:10.1371/journal.pmed.1002945)
Supplement: S1 Table — (DOCX) [file pmed.1002945.s002.docx]

**S1 Table Retinopathy and Maculopathy Grades and Retinopathy Severity Definitions**

| **Retinopathy** | **Description** |
| --- | --- |
| R0 (no visible retinopathy) | No diabetic retinopathy anywhere |
| R1 (mild) NPDR level 20 | Background diabetic retinopathy (BDR) The presence of at least one of any of the following features anywhere   - dot haemorrhages - microaneurysms - hard exudates - cotton wool spots - blot haemorrhages - superficial/ flame shaped haemorrhages |
| R2 (observable | Background diabetic retinopathy BDR – observable |
| background)  NPDR level 35, 43, 47 | -Four or more blot haemorrhages in one hemi-field only (Inferior and superior hemi-fields delineated by a line passing through the centre of the fovea and optic disc) |
| R3 (referable | Background diabetic retinopathy BDR - referable |
| background)  NPDR level 53 | Any of the following features:- Four or more blot haemorrhages in both inferior and superior hemi-fields  - Venous beading  - IRMA |
| R4 (proliferative)  NPDR level 61, 65, 71, 75, 80, 85 | Proliferative diabetic retinopathy PD:  Any of following features:   - Active new vessels - Vitreous haemorrhage |
| **Maculopathy** |  |
| M0 (no maculopathy) | No features within 2 disc diameters from the centre of the fovea sufficient to qualify for M1 or M2 as defined below |
| M1 (observable) | Lesions as specified below within a radius of > 1 but <2 disc diameters from the centre of the fovea  – Any hard exudates |
| M2 referable | Lesions as specified below within a radius of 1 disc diameter of the centre of the fovea  -Any blot haemorrhages  -Any hard exudates |
| **Retinopathy Severity** |  |
| No DR | R0M0 |
| Mild DR | R1M0 |
| Moderate DR | R2 and/or M1 |
