# Supplementary material for: Use of personalised risk-based screening schedules to optimise workload and sojourn time in screening programmes for diabetic retinopathy: A retrospective cohort study
Source: PLoS Med. 2019 Oct 17;16(10):e1002945. doi: 10.1371/journal.pmed.1002945 (PMC6797087; doi:10.1371/journal.pmed.1002945)
Supplement: S2 Table — (DOCX) [file pmed.1002945.s003.docx]

# S2 Table Strobe checklist

STROBE Statement—Checklist of items that should be included in reports of ***cohort studies***

|  | Item No | Recommendation | Page No |
| --- | --- | --- | --- |
| **Title and abstract** | 1 | (*a*) Indicate the study’s design with a commonly used term in the title or the abstract | Title page 1  Page 2 |
|  |  | (*b*) Provide in the abstract an informative and balanced summary of what was done and what was found |  |
| Introduction | | | |
| Background/rationale | 2 | Explain the scientific background and rationale for the investigation being reported | Intro paras 1,2,3 |
| Objectives | 3 | State specific objectives, including any prespecified hypotheses | Intro para 4 |
| Methods | | | |
| Study design | 4 | Present key elements of study design early in the paper | Mat & methods analysis para 1 |
| Setting | 5 | Describe the setting, locations, and relevant dates, including periods of recruitment, exposure, follow-up, and data collection | Mat & methods para 1 |
| Participants | 6 | (*a*) Give the eligibility criteria, and the sources and methods of selection of participants. Describe methods of follow-up | Mat& methods para 1 &2 |
|  |  | (*b*) For matched studies, give matching criteria and number of exposed and unexposed |  |
| Variables | 7 | Clearly define all outcomes, exposures, predictors, potential confounders, and effect modifiers. Give diagnostic criteria, if applicable | Mat & Methods para 3 retinopathy grades and  S1 Text |
| Data sources/ measurement | 8* | For each variable of interest, give sources of data and details of methods of assessment (measurement). Describe comparability of assessment methods if there is more than one group | Mat & Methods para 3 retinopathy grades and S1 Text |
| Bias | 9 | Describe any efforts to address potential sources of bias | S1 Text |
| Study size | 10 | Explain how the study size was arrived at | Mat & methods para 1 |
| Quantitative variables | 11 | Explain how quantitative variables were handled in the analyses. If applicable, describe which groupings were chosen and why | Mat & Methods para 3 retinopathy grades and S1 Text. |
| Statistical methods | 12 | (*a*) Describe all statistical methods, including those used to control for confounding | a) Mat & methods para 4 and S1 Text |
|  |  | (*b*) Describe any methods used to examine subgroups and interactions | b) mat & methods para 5 statistical methods c) Mat & methods para 4 imputation |
|  |  | (*c*) Explain how missing data were addressed |  |
|  |  | (*d*) If applicable, explain how loss to follow-up was addressed | d) not applicable  e)S1 Text differing screening schedules plus shiny app |
|  |  | (*e*) Describe any sensitivity analyses |  |
| Results | | |  |
| Participants | 13* | (a) Report numbers of individuals at each stage of study—eg numbers potentially eligible, examined for eligibility, confirmed eligible, included in the study, completing follow-up, and analysed | Results para 1 and Table 1 |
|  |  | (b) Give reasons for non-participation at each stage | b) not applicable full population evaluated  c) not needed full population evaluated |
|  |  | (c) Consider use of a flow diagram |  |
| Descriptive data | 14* | (a) Give characteristics of study participants (eg demographic, clinical, social) and information on exposures and potential confounders | a) Table 1 |
|  |  | (b) Indicate number of participants with missing data for each variable of interest | b) all subjects had screening data available  c) relevant statistic is number of screenings see abstract para 2  methods para 2 |
|  |  | (c) Summarise follow-up time (eg, average and total amount) |  |
| Outcome data | 15* | Report numbers of outcome events or summary measures over time | Interval disease rate see Table 2 and Table 3 |
