# Supplementary material for: Use of personalised risk-based screening schedules to optimise workload and sojourn time in screening programmes for diabetic retinopathy: A retrospective cohort study
Source: PLoS Med. 2019 Oct 17;16(10):e1002945. doi: 10.1371/journal.pmed.1002945 (PMC6797087; doi:10.1371/journal.pmed.1002945)
Supplement: S3 Table — (DOCX) [file pmed.1002945.s004.docx]

**S3 Table Comparison of predictive models for Type 1 and Type 2 diabetes**

|  | **T1D** | | | **T2D** | | |
| --- | --- | --- | --- | --- | --- | --- |
|  | C- statistic | Test log-likelihood  (natural log units) | Info of discrimination (bits) | C- statistic | Test log-likelihood  (natural log units) | Info of discrimination (bits) |
| (1) DR Grades only | 0.757 [0.738; 0.775] | 0.0 | 0.8 | 0.778 [0.767; 0.788] | 0.0 | 0.9 |
| (2) DR Grades + Age + Sex + Duration | 0.756 [0.737; 0.776] | 18.8 | 0.9 | 0.778 [0.767; 0.789] | 23.3 | 1.3 |
| (3) Full Model | 0.771 [0.753; 0.790] | 73.7 | 1.0 | 0.795 [0.785; 0.805] | 290.1 | 1.4 |
| (4) Backward selection | 0.771 [0.753; 0.790] | 72.6 | 1.0 | 0.794 [0.784; 0.805] | 287.1 | 1.4 |
| (5) DR Grades + Age + Sex + Duration + HbA1c + cholesterol | 0.770 [0.752; 0.789] | 71.4 | 1.0 | 0.794 [0.784; 0.804] | 271.8 | 1.4 |
| (6) Aspelund et al., 2011 | 0.755 [0.736; 0.774] | 2.1 | 0.9 | 0.787 [0.777; 0.797] | -72.2 | 1.3 |
| (7) DCCT, 2017 | 0.753 [0.734; 0.771] | -1.4 | 0.9 | 0.767 [0.756; 0.778] | -239.0 | 1.2 |

**Models contain the following variables:**

1. Penultimate two DR grades
2. Penultimate two DR grades and patients demographics i.e. age at grading, sex, and diabetes duration
3. Penultimate two DR grades and patients age at grading, sex, diabetes duration BMI, height, HbA1c, SBP, DBP, total cholesterol, HDL, visual acuity, eGFR, smoking status, statin and hypertensive drug use, and CVD status
4. Penultimate two DR grades and patients age at grading, sex diabetes duration, BMI, HbA1c, SBP, DBP, total cholesterol, and smoking status
5. Penultimate two DR grades and patients age at grading, sex diabetes duration, and selection constrained to retain only two additional variables- HbA1c, and total cholesterol retained
6. Previous DR grade, diabetes duration, HbA1c, SBP; as published in Aspelund et al., 2011
7. Previous DR grade, HbA1c; as published in DCCT, 2017
