# Supplementary material for: Use of personalised risk-based screening schedules to optimise workload and sojourn time in screening programmes for diabetic retinopathy: A retrospective cohort study
Source: PLoS Med. 2019 Oct 17;16(10):e1002945. doi: 10.1371/journal.pmed.1002945 (PMC6797087; doi:10.1371/journal.pmed.1002945)
Supplement: S1 Text — (DOCX) [file pmed.1002945.s005.docx]

## **S1 Text Details of methods**

## Retinopathy grades for modelling

For modelling, the grades at each examination were transformed by taking the sum and the difference of the R grades for each eye and the sum and the difference of the M grades. Taking the sum and the difference of two variables with equal variance transforms them to uncorrelated variables. Linear and quadratic terms for the R grades were included in the model. This allows for a more granular way of modelling prior states.

## Building the predictive model

A generalised linear model with complementary log-log link function to allow for interval censoring was used to model the transition to referable DR from the second screening episode. The models were fitted to a training dataset, containing a randomly selected 70% of individuals, and their predictive performance was evaluated on a test dataset, containing the remaining 30%. To choose the most predictive model of DR progression, a number of different models were fitted using the training dataset of this study (S3 Table). A base model containing previous penultimate two grades only (1), grades and demographics (2) grades, demographics and all risk factors available and previously reported in the literature associated with retinopathy (3) Model 3 but using backward selection for the clinical risk factors (4) Model 3 but with a constrained to select at most two variables on top of prior grade and two previously published models (6 and 7) The test log-likelihood was used to evaluate the strength of evidence. To quantify the increment in predictive performance, we used the C-statistic and also the expected information for discrimination Λ, measured in bits[1]. For quantifying increments in predictive performance, Λ is more interpretable than the C-statistic. Analyses were conducted using *R* version 3.3.3.

## Defining Screening Schedules

The best fitting model was used on the data to calculate for each patient the hazard rate of transitioning to referable DR at different time intervals, and to derive screening schedules. Two parameters were used to design screening schedules: (1) detection rate and (2) personalized/stratified schedules. The *detection rate* is the number of DR photographs graded as “referable” out of the screenings performed annually. It is thus the accepted risk of having developed referable DR at the time of the next screening. For initial analysis the detection rate was set equal to the overall detection rates expected in the current program: 2.4% for T1D, 0.6% for T2D. Screening schedules can be designed to be *personalized, or stratified* by the prior DR grade. For a personalized optimal screening schedule, the hazard function from the model was used to set a screening interval for each individual so that the probability of detecting referable DR in that individual equates the target detection rate. For a stratified schedule, one interval for each prior-DR-stratum specific interval was computed such that the average probability of transition to referable DR across all patients in each stratum by the end of that interval equated to the chosen risk threshold. Essentially, this is a schedule that maintains the prior DR strata but assigns screening intervals to each stratum that are risk based. This was done iteratively by computing the probability of transition averaged across all patients and choosing the interval at that time where the averaged probability equated the accepted detection rate.

## Evaluating Screening Schedules

Alternative screening schedules were compared in terms of the annual number of screens required, and the estimated average sojourn time in referable DR. Number of screenings required annually was quantified by computing the number of screenings needed per year based on each patients predicted interval and summing across all patients (personalised optimal schedule), or by computing the number of screenings needed per year in each stratum and multiplying by the number of patients in each stratum (stratified optimal schedule). Sojourn time in referable DR describes the time between onset of the disease and the time of detection at screening. To estimate the average sojourn time we used simulation. From the risk prediction model, we obtain a fitted value for the hazard rate *λ_i_* in the *i*th individual. For the detection rate to be *P* the length of the screening interval *t_i_* for that individual is

$$t_{i}=\frac{-log(1-P)}{\lambda_{i}}$$

We sampled failure times of individuals as exponentially distributed with parameter *λ_i_* for the *i*th individual.

# Reference

1 McKeigue P. Quantifying performance of a diagnostic test as the expected information for discrimination: Relation to the C-statistic. Statistical Methods in Medical Research. 2018;096228021877698.
